# Accelerating single molecule localization microscopy through parallel processing on a high‐performance computing cluster

**DOI:** 10.1111/jmi.12772

**Published:** 2018-12-03

**Authors:** I. MUNRO, E. GARCÍA, M. YAN, S. GULDBRAND, S. KUMAR, K. KWAKWA, C. DUNSBY, M.A.A. NEIL, P.M.W. FRENCH

**Affiliations:** ^1^ Photonics Group Physics Department, Imperial College London London U.K.; ^2^ Northwest Institute of Nuclear Technology Xi'an Shaanxi P.R. China; ^3^ The Francis Crick Institute London U.K.; ^4^ Centre for Pathology Imperial College London London U.K.

**Keywords:** Automated image analysis, high‐performance computing, super‐resolved microscopy

## Abstract

Super‐resolved microscopy techniques have revolutionized the ability to study biological structures below the diffraction limit. Single molecule localization microscopy (SMLM) techniques are widely used because they are relatively straightforward to implement and can be realized at relatively low cost, e.g. compared to laser scanning microscopy techniques. However, while the data analysis can be readily undertaken using open source or other software tools, large SMLM data volumes and the complexity of the algorithms used often lead to long image data processing times that can hinder the iterative optimization of experiments. There is increasing interest in high throughput SMLM, but its further development and application is inhibited by the data processing challenges. We present here a widely applicable approach to accelerating SMLM data processing via a parallelized implementation of ThunderSTORM on a high‐performance computing (HPC) cluster and quantify the speed advantage for a four‐node cluster (with 24 cores and 128 GB RAM per node) compared to a high specification (28 cores, 128 GB RAM, SSD‐enabled) desktop workstation. This data processing speed can be readily scaled by accessing more HPC resources. Our approach is not specific to ThunderSTORM and can be adapted for a wide range of SMLM software.

**Lay Description:**

Optical microscopy is now able to provide images with a resolution far beyond the diffraction limit thanks to relatively new super‐resolved microscopy (SRM) techniques, which have revolutionized the ability to study biological structures. One approach to SRM is to randomly switch on and off the emission of fluorescent molecules in an otherwise conventional fluorescence microscope. If only a sparse subset of the fluorescent molecules labelling a sample can be switched on at a time, then each emitter will be, on average, spaced further apart than the diffraction‐limited resolution of the conventional microscope and the separate bright spots in the image corresponding to each emitter can be localised to high precision by finding the centre of each feature using a computer program. Thus, a precise map of the emitter positions can be recorded by sequentially mapping the localisation of different subsets of emitters as they are switched on and others switched off.

Typically, this approach, described as single molecule localisation microscopy (SMLM), results in large image data sets that can take many minutes to hours to process, depending on the size of the field of view and whether the SMLM analysis employs a computationally‐intensive iterative algorithm. Such a slow workflow makes it difficult to optimise experiments and to analyse large numbers of samples. Faster SMLM experiments would be generally useful and automated high throughput SMLM studies of arrays of samples, such as cells, could be applied to drug discovery and other applications. However, the time required to process the resulting data would be prohibitive on a normal computer.

To address this, we have developed a method to run standard SMLM data analysis software tools in parallel on a high‐performance computing cluster (HPC). This can be used to accelerate the analysis of individual SMLM experiments or it can be scaled to analyse high throughput SMLM data by extending it to run on an arbitrary number of HPC processors in parallel. In this paper we outline the design of our parallelised SMLM software for HPC and quantify the speed advantage when implementing it on four HPC nodes compared to a powerful desktop computer.

## Introduction

Super‐resolved microscopy (SRM) techniques, including structured illumination approaches (Gustafsson, 2000; Gustafsson, 2005), stochastically switched single molecule localization techniques such as photo activated localization microscopy (PALM) (Betzig *et al*., 2006; Hess *et al*., 2006) and stochastic optical reconstruction microscopy (STORM) (Rust *et al*., 2006), and RESOLFT (Hofmann *et al*., 2005) techniques such as stimulated emission depletion microscopy (Hell & Wichmann, 1994; Klar *et al*., 2000) are transforming optical microscopy and providing new opportunities to study biology at scales below the classical diffraction limit. Although the early commercial implementations of SRM were relatively expensive compared to established optical microscopy instrumentation, there have been significant advances towards increasing access to SRM, e.g. in terms of lower cost excitation sources and detectors and openly shared data analysis tools.

Single molecule localization microscopy (SMLM) techniques, such as STORM and PALM, have relatively simple requirements for instrumentation, making them accessible to a broad spectrum of researchers. They utilize sequential activation and localization of ‘switchable’ fluorophores to create super‐resolved images, where the resolution is primarily determined by the ability to localize individual emitters. During the image acquisition, a subset of fluorophores is stochastically activated to a fluorescent state at any given time, such that the position of each fluorophore can be determined with high precision by determining the centre of the recorded intensity distribution. Activation and deactivation can be realized in many ways, e.g. by photoswitching fluorophores to emit in the detection band or otherwise, by photobleaching to terminate emission or by utilizing appropriate chemical buffers to facilitate reversible photoswitching of fluorophores in and out of dark states, as demonstrated in the technique described as dSTORM (Heilemann *et al*., 2008).

SMLM can particularly benefit from the use of low‐cost, high power, multimode laser diodes (Kwakwa *et al*., 2016) and low‐cost CCD (Holm *et al*., 2014) or CMOS cameras (Diekmann *et al*., 2017; Ma *et al*., 2017), which can be used with existing wide‐field fluorescence microscopes or with low‐cost custom‐built microscope frames [1212]. Access to SRM capabilities for academic researchers is further enhanced by freely available SMLM data processing tools, such as those listed in http://bigwww.epfl.ch/smlm/software/index.html. Although all of these tools will enable SMLM, there are different trade‐offs in performance and these have been elegantly summarized in Sage *et al*. (2015) for many of the most prevalent SMLM software tools.

SMLM techniques are increasingly incorporated in cell biology and other research programmes where they routinely provide image resolutions better than 50 nm. Automated SMLM could provide powerful readouts of assays for drug discovery or systems biology and extending SMLM to higher throughput is an exciting prospect, recognizing the importance of robust statistical analysis over many samples and noting the potential to average over labelling artefacts. However, SRM techniques are generally slower than conventional intensity imaging techniques and the concomitant data processing presents a significant challenge. Even for manual microscopy experiments, SMLM data sets can exceed 50 GB per field of view, particularly for 3D image data with large (120 × 120 μm) fields of view (FOV) (Kwakwa *et al*., 2016), for which the data processing can require 10's of minutes to hours on a standard desktop computer.

To date, there has been considerable work to increase SMLM data processing speeds. Typically, SMLM software is run on relatively powerful multicore desktop computer workstations, often integrated with the SMLM laboratory instrumentation. Although the SMLM data processing speed may be increased by upgrading the computer workstation, this provides diminishing return on investment once a configuration like that discussed below is reached [i.e. 28 cores, 128 GB RAM, graphics processing unit (GPU) and SSD‐enabled] and many laboratories will use lower performance computers. Significant effort has been directed towards optimizing code and computing performance of SMLM data processing software (Wolter *et al*., 2012) and GPUs) have been utilized for faster processing (Smith *et al*., 2010; Brede & Lakadamyali, 2012; Wang *et al*., 2012; Kechkar *et al*., 2013; Li *et al*., 2018). New, faster, localization algorithms have been devised, for example moving from the early SMLM approaches based on iterative fitting of sparse emitters to Gaussian profiles (e.g. Betzig *et al*., 2006; Rust *et al*., 2006) to algorithms able to fit higher densities of emitters with overlapping point spread functions (Holden *et al*., 2011; Wang *et al*., 2012; Zhu *et al*., 2012) to noniterative localization techniques (e.g. Henriques *et al*., 2010; Yu *et al*., 2011; Parthasarathy, 2012; Liu *et al*., 2013; Ma *et al*., 2015; Martens *et al*., 2018). After trying a number of different SMLM software tools to analyse our large (120 × 120 μm) field of view dSTORM data sets (often > 50 GB), we found ThunderSTORM (Ovesný *et al*., 2014) with iterative fitting of SMLM data to Gaussian point spread function (PSF) to provide the most useful combination of functionality and processing speed, noting that the benchmarking in Sage *et al*. (2015) supported this choice.

Higher throughput automated SMLM has already been demonstrated in pioneering work using PALM (Holden *et al*., 2014), dSTORM and DNA PAINT (Beghin *et al*., 2017) and could be beneficial for many SRM‐based studies but the image data processing presents challenges with respect to long processing times that scale with the imaging throughput achieved. For the high throughput dSTORM reported in Beghin *et al*. (2017), the authors argued that postprocessing the SMLM data would lead to unacceptable delays in the workflow and so they acquired and processed their dSTORM data on‐the‐fly, making the use of rapid GPU accelerated analysis software (Kechkar *et al*., 2013). Eight hours were required to image 96 cells in 96 wells and they observed that the buffers used to induce the fluorophore blinking led to degradation of the imaging performance over time (with ∼10 h being a practical limit for a dSTORM experiment). This limited the number of cells that could be imaged in a screen. For many high throughput applications, it would be important to increase the numbers of cells imaged per well. This could be addressed by imaging more cells in larger FOV and we note that the use of multimode optical fibres to efficiently deliver 100 s mW of excitation power from low‐cost multimode diode lasers to the sample enables dSTORM of FOV >100 × 100 μm to be routinely acquired (Kwakwa *et al*., 2016). Comparing this to the 20.5 × 20.5 μm FOV reported for (Beghin *et al*., 2017) suggests that high throughput dSTORM can be significantly accelerated if SMLM data are acquired for larger FOV (i.e. containing many more cells), but this would significantly increase the burden of data processing and so would increase the total acquisition time required if the SMLM data are processed on the fly. The large data volumes would also challenge the available on‐board GPU memory. We therefore conclude that higher throughput SMLM would benefit significantly from higher speed data postprocessing of the large data sets resulting from SMLM data acquisition across large FOV.

To accelerate the postprocessing of SMLM data, we further parallelized an open source SMLM image analysis tool, ThunderSTORM, by running multiple instances simultaneously on subsets of the data on a high‐performance computing (HPC) cluster, which provides a cost‐effective means to accelerate SMLM data processing that can be readily scaled to utilize additional HPC resource for high throughput applications. Here, we demonstrate its application to exemplar experimental dSTORM data, although we note that the image processing tools we discuss below could be applied to any SMLM analysis method that can be easily divided into smaller independent processing tasks. Our implementation with ThunderSTORM utilizes the open‐source Bio‐Formats library (Linkert *et al*., 2010) to prepare the data for parallel processing and could be adapted for any SMLM analysis software available as an ImageJ (Schindelin *et al*., 2015) plug‐in that analyses subsets of the data independently. Relatively minor modifications to our code would enable the chosen SMLM analysis plug‐in to be run on HPC resources without modification to the plug‐in and with minimal manual intervention.

One potential disadvantage of using centralized HPC resources is the use of queuing protocols that do not return immediate results. Since we are also interested in realising SMLM analysis in close to real‐time, such that the results can inform ongoing imaging experiments, we have implemented HPC ThunderSTORM on a dedicated four‐node cluster (with 24 cores and 128 GB RAM per node) that provides a priority queue for immediate processing. This can be relatively cost‐efficient compared to purchasing high end desktop workstations, as well as providing sufficient RAM to accommodate large SMLM data sets and the possibility to readily scale to further nodes as required. We have not utilized GPU processing, noting that, while GPU nodes are available for HPC clusters, they are relatively expensive and require more specific programming expertise. Although this could be addressed in future extensions of our work, we speculate that scaling up the SMLM analysis rate by using more standard HPC processing nodes may be more cost‐effective than investing in arrays of GPU nodes.

We were also interested to compare the processing speed of SMLM data processing using iterative fitting of emitters to Gaussian profiles with a noniterative localization technique and have taken advantage of the recent availability of the ThunderSTORM plug‐in providing noniterative phasor‐based localization (Martens *et al*., 2018) for SMLM to provide such a comparison. Although the resulting super‐resolved images are not the same as those generated by iterative fitting, the phasor localization approach provides images relatively rapidly for both 2D and 3D SMLM data when implemented on a reasonably fast desktop computer – even for large data sets. If this is implemented on the data acquisition computer, there would be no delay in copying data and the phasor‐localization approach could provide ‘preview images’ feedback to SMLM users while their experiments are still underway.

## Parallelization of SMLM analysis for HPC

**Figure 1:**
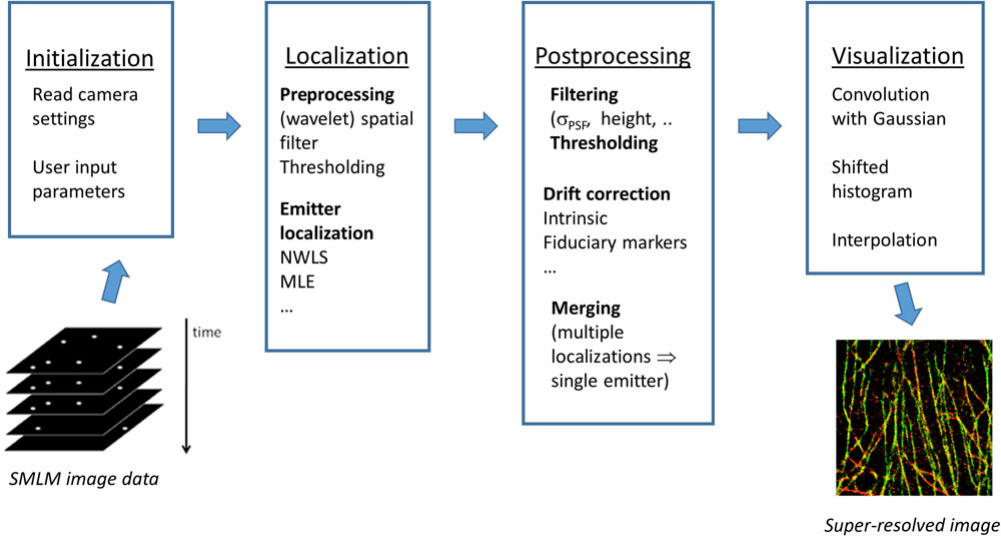
Schematic of SMLM data analysis workflow.

Our software consists of a suite of Bash Linux shell scripts and macros written in the ImageJ macro language. It has been developed and tested running the open‐source ImageJ plugin ThunderSTORM (Ovesný *et al*., 2014; https://zitmen.github.io/thunderstorm/) on an HPC cluster that uses open‐source PBS Professional (http://www.pbspro.org/) for job scheduling and workload management. In general, the data processing pipeline for SMLM can be considered in four key stages, as depicted in Figure 1, of which the second and most intensive is that of localizing each emitter in each frame of the acquired image data. This is followed by a postprocessing stage to apply drift correction and filters to the localization data set, which may require manual user intervention to determine appropriate settings or which can be automated, and then by an image visualization stage that renders the processed localization data set as super‐resolved image(s). Where the SMLM data processing requires more time than the data acquisition, it is often convenient to analyse multiple SMLM data sets ‘offline’ after an image acquisition session. This could be appropriate for manual microscopy with large FOV or for an automated high throughput SMLM system. Accordingly, we have developed two workflows for SMLM processing: ‘Parallel mode’ and ‘Batch mode’, which each run using a single set of scripts, in order to simplify software maintenance.

### Parallel mode

This mode has been developed to enable users to analyse a large SMLM data set as quickly as possible by sharing the localization task between multiple HPC nodes. The parallelization strategy assumes that the processing of each frame in the raw data to find the localizations is independent of all the other frames. Subsets of the raw image data can therefore be processed independently and in parallel by each available node in the cluster. This is analogous to the approach taken internally by the ThunderSTORM plug‐in to more effectively utilize the multiple processing cores available in a single computer node. In practice, however, we found that a single instance of ThunderSTORM running on a node typically still does not fully utilize all the available CPU resources and so further improvements in processing time can be obtained by further subdividing the data and running multiple instances of ThunderSTORM in parallel on each node. This parallel approach can provide a considerable improvement in the intensive localization processing stage. The tables of localizations obtained from each node are merged into a single file to be processed on a single node since the subsequent steps, such as postprocessing on the table of localizations and generating images, do not add significantly to the total processing time. For convenience, preview images can be automatically and rapidly generated from the filtered localization table to inform the user when optimizing both experimental and data analysis parameters. This could help users, e.g. to quickly judge the success of a given experimental protocol, or to decide whether further postprocessing is necessary or to identify features or regions in the field of view on which to concentrate the analysis.

### Batch mode

This mode is intended for use where a number of SMLM data sets are to be automatically processed with no manual user intervention, e.g. after acquisition of a z‐stack or an array of samples. In this mode, each available cluster node is used to sequentially process the SMLM data corresponding to a single FOV – from localization to postprocessing – thereby avoiding the overhead associated with the copying of the raw data to several different nodes. On each node, significant reductions in processing time can still be obtained by splitting the job among multiple instances of ThunderSTORM to more effectively utilize each node's processing resources.

### Postprocessing and visualization

After the raw localization data have been generated, it must be interleaved again if multiple ThunderSTORM instances are use, and then a number of processing steps can be applied, including thresholding, sigma filtering drift correction and combining multiple localizations occurring in subsequent frames that represent a single emitter. Note that the postprocessing stage is not parallelized and operates on the entire data set. These postprocessing steps might be selected manually, with the user trying different settings in order for the user to optimize setting for a final SMLM image. Alternatively, a predetermined set of settings could be used to automate the postprocessing steps, as would be appropriate if a large number of images were being analysed in batch mode.

The visualisation step entails rendering the corrected (i.e. postprocessed) localization data to produce the desired super‐resolved image. There are a number of approaches to visualization available in ThunderSTORM, including convolving the localization table with a Gaussian function, ‘average shifted histogram’, ‘scatter plot’ and ‘histograms’. Typically, this computation is much faster than the localization and it is not worth the overhead to parallelize this task between multiple HPC cores. However, given that this stage makes little advantage of available parallel CPU resources on a single node, it would be possible to generate multiple super‐resolved images by running visualization jobs in parallel on a single node using different jobs to implement different approaches or settings applied to the same SMLM data set.

## Methodology

Our approach utilizes the ImageJ (https://imagej.nih.gov/ij/) open‐source image‐processing platform with the Bio‐formats ImageJ plug‐in, developed as part of the Open Microscopy Environment, providing the capability to load subsets of data from the raw SMLM data files. Specifically, the Fiji (Schindelin *et al*., 2012) distribution of ImageJ was installed along with the Bio‐formats and ThunderSTORM plug‐ins. Since it proved impossible to run this combination ‘headless’, i.e. without a graphics environment, the Virtual Network Computing graphical desktop sharing system was used to provide a headless X‐server (xvnc) on each node of the HPC cluster.

All software is available from https://github.com/ImperialCollegeLondon/HPC_STORM.

### Load balancing

In order to achieve the shortest processing time in parallel mode, it is important to share the processing work equally between the available HPC cluster nodes since the final table of localizations cannot be produced until all nodes have finished their subsets of the SMLM data. If the work is unevenly allocated, then the other nodes may remain idle until the ‘slowest’ node (i.e. that which has been allocated the most processing‐intensive task) finishes. One possible approach to load balancing is to breakdown the localization task to a large number of smaller jobs that process subsets of sequential frames and allow the PBS Professional queuing system to allocate these jobs as processing nodes become available. Unfortunately, there is an overhead each time a new job is scheduled, so dividing the SMLM data between a large number of small jobs would be inefficient. A more efficient approach would be to allocate each node with a single job of the same processing burden, but then it can be difficult to accurately predict the processing‐time required to determine the localizations, since this depends strongly on the number of localizations per frame, which can change over the acquisition, e.g. due to photobleaching. Instead the SMLM data are divided into interleaved data sets (i.e. for our four‐node HPC cluster, node one processes frames 1, 5, 9, 13, …, node two processes frames 2, 6, 10, 14, … etc.). Using this approach, the distribution of localizations across the different frame sets is comparable and so too are the parallel job execution times. After localization is completed, the localization results are merged by interleaving the localization tables using the ‘awk’ and ‘sort’ commands in UNIX before passing the data back to the ThunderSTORM plug‐in for filtering, drift correction and visualization.

**Figure 2:**
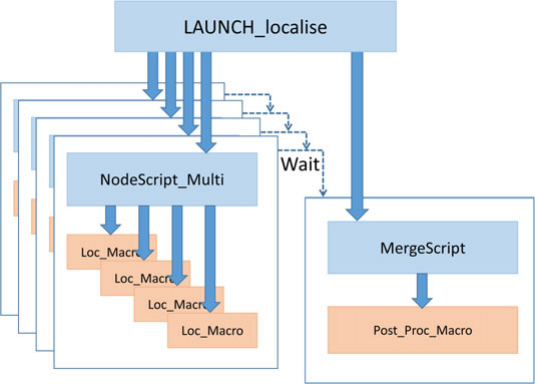
Schematic of data flow for parallel HPC analysis of SMLM data.

Figure 2 shows the flow of the data analysis. Typical input SMLM data can be several tens of gigabytes in size, depending on the image acquisition parameters, whereas the output data from the localization stage are only of order one gigabyte in total, depending on the number of localizations found, and the final rendered image results are only a few megabytes in size. The data bottle‐neck in the system is the transfer of the input SMLM data to the individual processing nodes, the speed of which is dependent on the network infrastructure available. Each HPC node is sent the data it will process, which are stored on its local HDD for processing.

### Software structure

All the scripts and ImageJ macros are installed in a subdirectory of the user's home directory on the HPC cluster (called ‘Localization’). The data SMLM can be located in any directory to which the user has access for scp (secure copy). For 3D processing, the required calibration file must be located in the same directory as the raw SMLM data file.

The raw SMLM data file can be in any file format that the open source Bio‐Formats library can read as a time‐series. However, testing and development has been carried out using the OME‐TIFF files generated by the Micro‐Manager Open Source Microscopy Software (Edelstein *et al*., 2010; https://micro-manager.org/). All output files, including .csv files containing both raw and automatically postprocessed localization tables, preview images and a log file describing all the processing steps, are then written back to the same directory where the input files were located.

The jobs are launched when the user runs a BASH script called ‘LAUNCH_localise.sh’, which takes the path to the input data as an argument. Adding a second argument with the name of a calibration file will trigger 3D processing. Finally, adding a ‘‐b’ argument will cause these data to be processed in batch mode on a single processing node, leaving the other nodes available to process further images.

**Figure 3:**
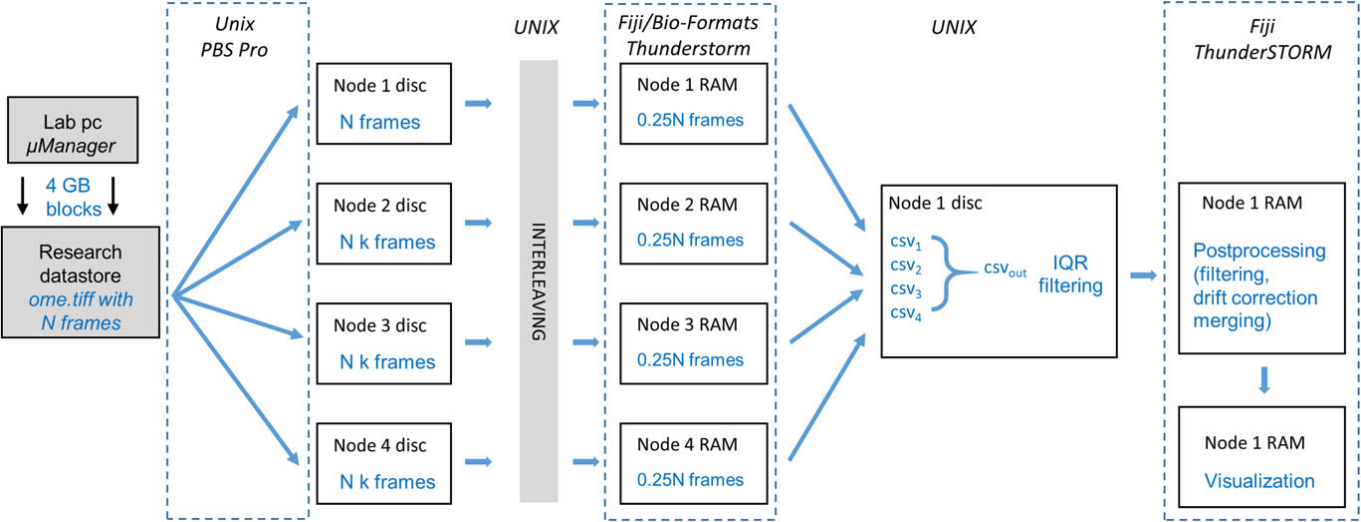
Schematic of software structure for HPC SMLM data analysis.

This ‘LAUNCH_localise.sh’ script then submits two jobs to the PBS Professional queue. The first job script submitted is ‘NodeScript_Multi.pbs’, which runs as an array job in parallel on one or more nodes, as requested by the user. On each node, this script can run one or more parallel instances of FIJI/ThunderSTORM, each performing the required localization calculations on a subset of the input SMLM data. On completion of all elements of this array job, the remaining merging and postprocessing job, MergeScript.pbs, then runs on a single node, combining the outputs of the array jobs and loading them back into a single instance of Fiji/ThunderSTORM to perform any required postprocessing and visualization tasks. A schematic of the processing scripts is shown in Figure 3.

#### Processing (array) job

This array job consists of a script called ‘NodeScript_Multi’ that initially copies the input data to the local disc. It then runs an ImageJ macro ‘Loc_Macro’ in the required parallel instances of Fiji, which uses ThunderSTORM to create a table of localizations for a subset of the input SMLM data, having first set up ThunderSTORM with the appropriate camera settings, determined using the metadata in the SMLM data file. The input data are shared among the *n* different instances of ThunderSTORM running on the different nodes by using the Bio‐Formats plugin to load every *n*th image in the stack, with each instance of ThunderSTORM using a different starting image in the series. In this way, the load is balanced among the different instances of Thunderstorm, as each has a broadly similar data set to process. The table of results from the localization process is written as a .csv file, to the local disc. Two text files, a config file detailing which subset of frames has been processed and a log file detailing all the steps in a human‐readable form, are also created. Control is then returned to the calling script (NodeScript_Multi), which copies all output files to a temporary subdirectory in the user's home directory, accessible from all nodes of the cluster. Finally, all temporary data stored on the node are automatically deleted once the job ends.

#### Merge and postprocessing

This job consists of a script called ‘MergeScript’, which initially recombines the separate .csv files from the user's temporary home directory using Unix utilities ‘awk’ and ‘sort’ to arrange the data into the original image frame order, which is important for some subsequent postprocessing utilities in Thunderstorm. It also concatenates all the log files into a single file and then runs an ImageJ macro ‘Post_Proc_Macro’ that uses ThunderSTORM to open the combined .csv file, to perform any required extra filtering and to generate a preview image if required.

Automatic filtering can be implemented to remove unphysical localization results with intensity values (number of photons) less than one and further filtering can remove localizations with uncertainty in *z* of greater than 500 nm for 3D localizations. We have also implemented an optional filtering step in the post processing ImageJ macro that uses the UNIX ‘awk’ utility to quickly estimate the centile points in the sigma distribution and then uses these values in ThunderSTORM to delete localizations whose width (sigma) lies outside the 10th–75th intercentile range. The optional filtering step for *x*–*y* drift correction provided within ThunderSTORM has also been incorporated in our code. If this drift correction is selected as one of the postprocessing steps, then ThunderSTORM also generates a graph that summarizes the results of this drift correction and this is saved as one of the outputs. The optional postprocessing steps are configurable by changing a string passed in from the LAUNCH_localise script. This mechanism makes it straightforward to include further steps in the automated postprocessing of the table of localizations as offered by ThunderSTORM. More sophisticated postprocessing may include thresholding and filtering, usually according to manually set criteria, e.g. with respect to width and height of localization peaks, to reduce the impact of noise. A merging step to combine multiple localizations occurring in subsequent frames to represent a single emitter may be applied, enabling duplicate localizations at the same position but different time points to be excluded for single molecule counting applications. The postprocessed localization table is also saved to disc at this point, again as a .csv file with a file name including the postprocessing configuration string described above.

If a preview image has been requested, the last action performed by ‘Post_Proc_Macro’ is to use the postprocessed table of localizations to generate this using the average shifted histogram method. For 3D data sets, we additionally take the 3D stack output from ThunderSTORM and combine the data using standard ImageJ operations into a 2D image, colour coded for average *z* position in each pixel. The final rendered output is written to disc with a filename containing either the string ‘2D’ or ‘3D’ as appropriate.

Finally, MergeScript copies all output files back to the directory in which the input file was originally located, under a subdirectory named after the unique system job number. This makes it straightforward to create multiple postprocessing jobs, each performing different postprocessing options as required. Again, all temporary data stored on the node are automatically deleted once the job ends.

### Experimental single molecule localization microscopy

To illustrate the performance of the HPCS‐MLM data processing, 2D and 3D STORM data were acquired using an ‘easySTORM’ microscope system described in Kwakwa *et al*. (2016). SMLM images were acquired using dSTORM of fixed cells, in which alpha tubulin was labelled with Alexa Fluor 488 and/or Acetyl alpha‐tubulin with Alexa Fluor 647.

#### Cell culture

Mouse embryonic fibroblasts (NIH‐3T3) (ATCC, CRL‐1658, USA) were seeded at a density of 20 000 cells per well in 8 well Labtek chamber slides (NUNC, 7342062, Denmark) in 400 μL of Complete media [DMEM, supplemented with 10% fetal bovine serum (FBS) and 200 mM glutamine] and cultured under standard cell culture conditions (37°C, 5% CO_2_ humidified atmosphere) for 24 h. Cells were fixed with 4% paraformaldehyde in PBS for 10 min, permeabilized with permeabilization solution (PBS‐Triton X 0.05% for 10 min), blocked with Blocking solution (permeabilization solution supplemented with 10% FBS) and probed with primary antibodies for alpha tubulin (Sigma Aldrich, T8203, USA) 1:2000 dilution in Blocking solution and/or acetyl alpha‐tubulin Lys 40 Monoclonal 6–11B‐1 (ThermoFisher Scientific, 32–2700, USA) for 1 h at 37°C or overnight at 4°C in Blocking solution. After three washes with permeabilization solution, a secondary antibody conjugated with Alexa Fluor 488 at a dilution 1:2000 in Blocking solution (ThermoFisher Scientific, A‐21121, USA) and/or an antibody conjugated with Alexa Fluor 647 (ThermoFisher Scientific, A‐31571, USA) was added and incubated at room temperature for 20 min followed by three washes with permeabilization solution to remove excess unbound antibody. Samples were subsequently washed three times with phosphate buffer saline (PBS) at pH 8.7. Acetylation of alpha‐tubulin was induced by treating cells for 72 h with an inhibitor of deacetylases trichostatin‐A (Cayman Chemical, 89730, USA) at a final concentration of 100 nM.

#### Sample preparation for dSTORM

The dSTORM imaging buffer (hereafter STORM buffer) was made prior to imaging and consists of 50 mM mercaptamine (Sigma‐Aldrich, M6500, USA), 10 mM DL‐lactate (Sigma‐Aldrich, L1375, USA) in PBS pH 8.7. Samples were washed three times with 400 μL of STORM buffer. Subsequently, 10 μL of oxyrase enzyme (Sigma‐Aldrich, SAE0010, USA) was added to each sample. The chamber slide was sealed with Parafilm (Sigma‐Aldrich, P7793‐1EA, USA) and incubated 10 min at 37°C prior to imaging on the STORM microscope.

#### Image acquisition

Our easySTORM (Kwakwa *et al*., 2016) implementation of dSTORM utilizes a multimode laser diode source (Cairn Research Ltd, UK) in combination with an inverted microscope frame (Carl Zeiss GmbH, Axiovert 200, Germany) and a total internal reflection excitation beam coupling unit (OptoTIRF, Cairn Research Ltd, UK). 3D STORM is implemented using a cylindrical lens in the emission path to provide an astigmatic point spread function that enables axial localization of the fluorophores (Holtzer *et al*., 2007; Huang *et al*., 2008). The chamber slide was imaged using a 100x, 1.46 NA oil lens in an epifluorescence microscope (Carl Zeiss, Axiovert 200, Germany) with no autofocus that was configured for easySTORM (Kwakwa *et al*., 2016) (using an OptoTIRF module, Cairn Research Ltd) with a sCMOS camera (Photometrics Prime 95B, UK). After identifying a suitable field of view, the sample was allowed to settle for 10 min to reduce thermal drift during acquisition.

To image structures labelled with Alexa Fluor 647, the cells were excited at 635 nm with an initial excitation intensity of 2500 μW/cm^2^ at the sample plane to activate fluorophore blinking and then power was decreased to ∼50% during image acquisition using a camera integration of 30 ms per frame. To image structures labelled with Alexa Fluor 488, excitation at 462 nm was initially set to 1045 μW/cm^2^ and then reduced to ∼50% during image acquisition. Axial localization for 3D STORM was implemented using a cylindrical lens of 1 m focal length in the emission light path, placed at a distance of approximately 40 mm in front of the camera chip.

#### Data processing

The SMLM data analysis was undertaken on either a dedicated four‐node priority queue on an HPC cluster or a desktop computer running Windows 7. Each node of the HPC cluster runs two Intel(R) Xeon(R) CPU E5‐2650 v4 @ 2.20 GHz processors, each of which has 12 physical cores (corresponding to 24 logical cores with hyperthreading), resulting in a total of 48 available logical cores on each node. Each node has 128 GB allocated RAM and local hard disc drive. The desktop computer used for the comparison runs an Intel(R) Xeon(R) CPU E5‐2690 v4 @ 2.60 GHz. This processor has 14 physical cores (28 logical cores) and 128 GB RAM. In addition to the higher clock speed, it is also equipped with a solid‐state drive for storage.

## Performance of HPC SMLM analysis

### Single molecule localization speed

**Figure 4:**
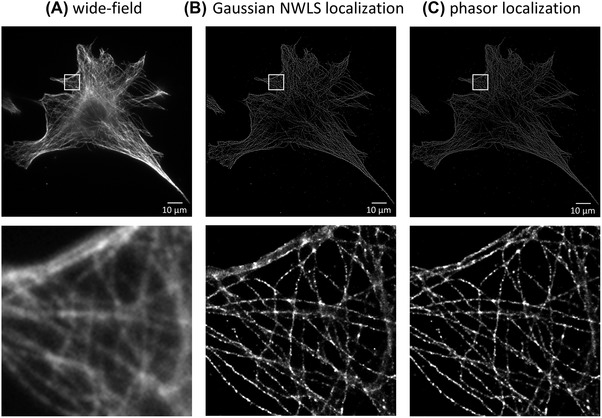
Images of acetylated tubulin in NIH‐3T3 a mouse embryonic fibroblast: (A) wide‐field fluorescence image; (B, C) automatically processed (averaged shifted histogram) visualization of Gaussian NWLS fitting localization table (B) and phasor‐based localization table (C), with lateral drift correction; (scale bar is 10 μm, inset images are 10 μm × 10 μm).

Figure 4 shows images reconstructed from a 2D dSTORM data set of an NIH‐3T3 mouse embryonic fibroblast with alpha tubulin labelled using antibodies conjugated to Alexa Fluor 647. The (13.4 GB, 5000 frames) SMLM data set was processed using ThunderSTORM with the HPC cluster scripts running in both parallel mode on a priority queue with four HPC nodes and on a single node (i.e. in batch mode), and also on the high specification desktop computer to which we had access. For comparison, we undertook the 2D localization processing stage using iterative fitting to a Gaussian profile and using phasor‐based localization. Figure 4(B) and (C) shows how the reconstructed images compare for these two localization techniques.

Table 1 presents the timings for key stages in the SMLM data processing. Timings for the parallelizable localization section have been separated from those of the postprocessing and visualization stages that are processed on a single node; both sections include timings for reading in and writing out of data, some of which also benefit from parallelization. Iterative nonlinear weighted least squares (NWLS) fitting to a Gaussian profile required 41 min 53 s on the desktop computer and 54 min 4 s on a single HPC node. The relative timings for phasor‐based localization were similar for the single HPC node and desktop PC (data not shown) and were approximately four times less than the time required for iterative fitting to a Gaussian profile. However, utilizing four nodes of the HPC cluster in batch mode would allow three further image data sets to be processed simultaneously in the same time – and this could be extended arbitrarily, e.g. for high throughput multiwell plate SMLM. When the four HPC nodes were used in parallel mode, the localization step was approximately 4x faster running a single job on each node, and over 11x faster when running four jobs on each of four nodes. In practice, the total SMLM data processing time also includes the time to copy the SMLM data to the analysis computer and any overheads or delays introduced by the job scheduling system used on the HPC cluster. These timings have been excluded from the comparisons presented here because they depend critically on the specific local computer network infrastructure and the implementation of the image data storage.

**Table 1:** Comparison of processing times with iterative fitting to Gaussian PSF and phasor‐ThunderSTORM for SMLM data analysis using the desktop computer and using one HPC node or the four‐node HPC cluster in parallel mode

File size (gigabytes)	13.4 GB (5000 frames)	
Desktop PC (14 cores)	Phasor	Gaussian NWLS
*Find localizations*	9 min 30 s	39 min 17 s
*Postprocessing: filtering lateral drift correction and visualization*	2 min 30 s	2 min 36 s
*Number of localizations (number after filtering)*	11 168 044 (11 093 056)	10 981 212 (10 981 146)
***Total processing time***	**12 min 00 s**	**41 min 53 s**

	Gaussian NWLS			
	One node	Four nodes	One node	Four nodes
HPC cluster	One job	One job/node	Four jobs	Four jobs/node
*Find localizations*	48 min 58 s	11 min 46 s	17 min 15 s	4 min 16 s
*Postprocessing: filtering, lateral drift correction and visualization*	5 min 06 s	5 min 14s	5 min 14 s	5 min 16 s
*Number of localizations (number after filtering)*	10 981 212 (10 981 146)	10 981 212 (10 981 146)	10 981 212 (10 981 146)	10 981 212 (10 981 146)
***Total processing time***	**54 min 04 s**	**17 min 00 s**	**22 min 29 s**	**9 min 32 s**

**Figure 5:**
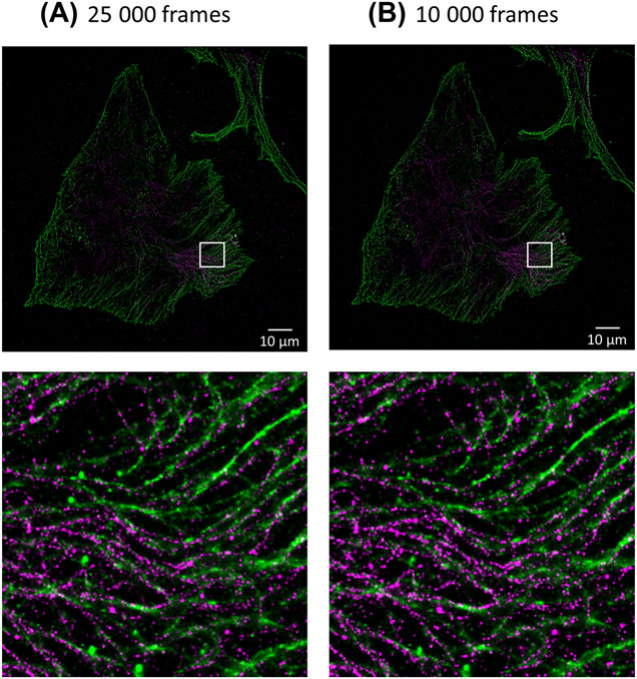
Reconstructed images (with expanded regions to show more detail) of alpha‐tubulin (green) and acetylated tubulin (red) in NIH‐3T3 Mouse Embryonic Fibroblasts generated by parallel HPC analysis using the 4 × 24‐core node cluster. Images reconstructed from (A) the full 25 000 frames and (B) a subset containing the first 10 000 frames of the acquired data. (Scale bar is 10 μm, inset images are 10 μm × 10 μm).

Figure 5 shows a two‐colour image reconstructed from a more challenging 2D, 2‐colour dSTORM data set of an NIH‐3T3 mouse embryonic fibroblast with alpha tubulin labelled with antibodies conjugated to Alexa Fluor 488 and acetylated tubulin labelled with antibodies conjugated to Alexa Fluor 647. The raw data comprise 25 000 frames comprising 67.3 GB of data contained in a set of 17 files for each channel. It will be seen in Table 2 that the relatively high specification desktop PC requires 163 min 31 s to process the full Alexa Fluor 488 data set compared to 24 min 40 s when using a parallelized four‐node HPC cluster for Gaussian NWLS processing. The total processing time could be readily reduced further by utilizing a larger number of HPC nodes. Using Phasor processing, the desktop PC processes the full data set in 41 min 24 s.

**Table 2:** Comparison of processing times of SMLM data represented in Figure 5 for different stages of SMLM data analysis using the desktop computer, and the four‐node HPC cluster in parallel mode 5

AF488 dSTORM (67.3 GB)	Four HPC nodes four jobs/node				Desktop PC	
Algorithm	Gaussian NWLS				Phasor	
Frames	25k	10k	25k	10k	25k	10k
*Find localizations*	16 min 34 s	10 min 18 s	159 min 07 s	122 min 57 s	37 min 36 s	20 min 26 s
*Postprocessing (filtering, drift correction and visualization)*	8 min 06 s	7 min 28 s	4 min 24 s	3 min 34 s	3 min 48 s	3 min 39 s
*Number of localizations (number after filtering)*	19 308 944 (19 308 355)	16 975 777 (16 975 196)	19 308 944 (19 308 355)	16 975 777 (16 975 196)	19 981 786 (19 546 283)	17 645 143 (17 269 087)
***Total processing time***	**24 min 40 s**	**17 min 41 s**	**163 min 31 s**	**126 min 31 s**	**41 min 24 s**	**24 min 05 s**

Note: Timings are shown for Gaussian NWLS fitting on both HPC and the Desktop PC, and for Phasor fitting on the Desktop PC. Each algorithm is applied to either the full 25 000 frames.

This large data set results from a large (∼125 × 125 μm) field of view of a densely labelled sample. Since the required SMLM data processing times are so long, it is interesting to consider time impact of processing only an early subset of the acquired frames in order to reduce the data analysis and storage requirements. Generally, the number of localizations decreases approximately exponentially with increasing frame number, although this can vary – particularly if the experimental parameters are adjusted during the acquisition. Since the earlier frames typically contain more of the localizations, it is worth considering whether the information gained from processing later acquired frames justifies the time and data storage costs. The impact of acquiring/processing fewer frames can vary from experiment to experiment and from sample to sample but acquiring significantly fewer than 25 000 frames will usually be a reasonable decision. To illustrate this, Figure 5 compares the reconstructed images for this sample when processing the full data set of 25 000 frames and when only processing the first 10 000 of the frames acquired in each channel – and indicates that there is not a significant reduction in quality when using only 10 000 frames. In the blue channel (timing results shown in Table 2), the first 10 000 frames contain 87% of the total localizations in the 25 000‐frame data set using Gaussian NWLS fitting, whereas in the red channel the proportion was 49%. For the timings shown in Table 2, the reduction in the processing time is less than the reduction in the number of frames processed, because there are more localizations to process in the earlier frames. However, the reduction in storage required for the 10 000‐frame data set (i.e. only 27 GB in the absence of data compression) is a significant benefit.

**Figure 6:**
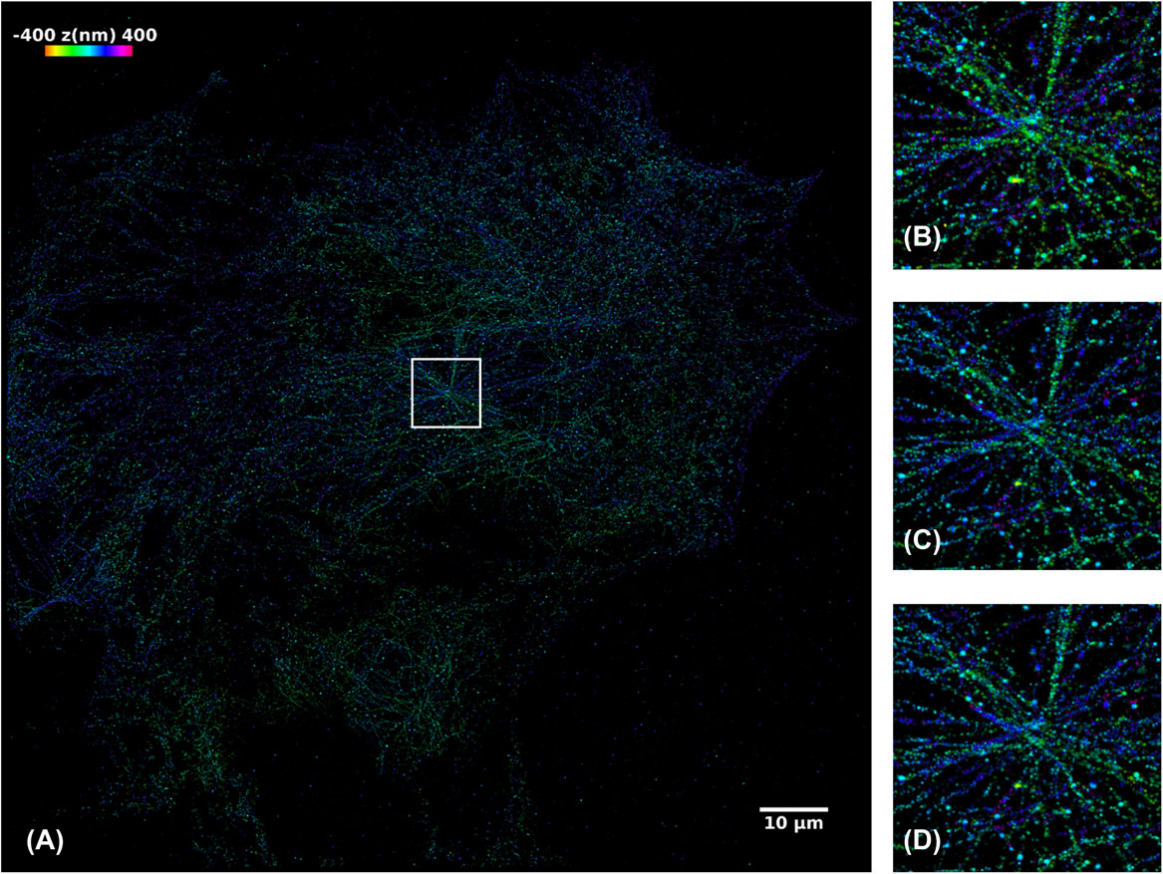
Depth‐resolved dSTORM images of NIH3T3 cell with alpha‐tubulin labelled with Alexa Fluor 647 reconstructed using ThunderSTORM with (A) 10 000‐frame data set (of 29 GB) reconstructed using (B) phasor‐based localization, (C) Gaussian nonlinear weighted least squares fitting and (D) Gaussian fitting using maximum likelihood estimation. Full size image (A) uses NLS and shows the 10 × 10 μm area zoomed for (C and D). (Scale bar 10 μm; depth colour scale: –400 to +400 nm).

A final comparison was made with a 3D dSTORM data set of a mouse embryonic fibroblast with alpha tubulin labelled with Alexa Fluor 647 conjugated antibodies. The raw data comprised 10 000 frames acquired with 30 ms camera integration and the data were analysed using phasor localization and using iterative fitting to a Gaussian profile with either NWLS fitting or maximum likelihood estimation (MLE). Note that MLE takes significantly longer to compute, even when distributed over many nodes and jobs – computation of Gaussian fitting using MLE was abandoned on the desktop PC after its run‐time exceeded 24 h. Figure 6 shows the resulting reconstructed images for this 3D SMLM data set with depth encoded as colour and Table 3 shows the data processing times required for the desktop PC and the four‐node HPC cluster with filtering to remove localizations indicating a large uncertainty in *z* (uncertainty output data only available for Gaussian fitting) and drift correction. The resulting images for Gaussian NWLS and MLE fitting are highly consistent with each other, whereas the image reconstructed from the phasor analysis shows small differences.

**Table 3:** Comparison of processing times for different stages of SMLM data using the desktop computer and using the four‐node HPC cluster in parallel mode

3D STORM (File size 10 000 frames, 29 GB)	Desktop PC Phasor fitting	Desktop PC Gaussian NWLS	HPC four nodes four jobs/node NWLS	HPC four nodes four jobs/node MLE
*Find localizations*	9 min 26 s	116 min 32 s	11 min 31 s	229 min 52 s
*Postprocessing: filtering, lateral drift correction and visualization*	3 min 27 s	3 min 33 s	4 min 29 s	4 min 18 s
*Number of localizations (number after filtering)*	8 764 013 (8 707 036)	8 677 386 (6 754 187)	8 677 386 (6 754 187)	8 683 023 (6 767 722)
***Total processing time excluding visualization***	**12 min 46 s**	**120 min 05 s**	**16 min 00 s**	**234 min 10 s**

## Conclusions

Motivated by the desire to develop a high throughput SMLM platform capable of analysing large data sets comprising multiple FOV of >120 × 120 μm, we have demonstrated a modular approach to SMLM analysis that can be implemented on an HPC cluster to provide acceleration of SMLM data processing through parallelization. In particular, Tables 1, 2, 3 show the reduction in data processing time for the critical SMLM localization step that is achievable with parallel processing using just four HPC nodes in parallel. With large SMLM data sets for complex images – or for arrays of images – this parallelization can easily be extended to utilize more HPC resource as required to scale over orders of magnitude of data volumes.

We note that our approach to parallel SMLM data processing on HPC nodes can take advantage of many speed enhancements due to improved hardware or software, noting that it can be rapidly implemented with any SMLM software tool compatible with Bio‐Formats that can analyse a subset of the data independently. For manual SMLM experiments, the overheads associated with transferring the data to an HPC cluster may outweigh the advantage gained by parallelizing the processing and this decision will depend on the local network infrastructure as well as the processing power of the local laboratory computer. The phasor‐based approach of Martens *et al*. (2018) is interesting since it is already available as a plug‐in to ThunderSTORM and could provide useful preview images, particularly if it can be implemented on the laboratory computer to which the SMLM data are acquired.

We believe that the acceleration of processing is important for higher throughput SMLM, where multiple replicates for each experimental condition may be imaged. If these SMLM data can be acquired with multiple FOV per well in 96 or 384 well plates, it is likely to be impractical to process these data on the fly. Since parallel processing of SMLM data can be scaled simply by increasing the number of HPC nodes utilized, we believe that the approach we present here can find wide application, particularly noting the ongoing reduction in HPC computing costs. Besides screening with SMLM‐based assays, for example, it would enable convenient analysis of SMLM data sets corresponding to many different image acquisitions, for example, enabling studies of time‐series SMLM data or for single particle averaging to combine SMLM data from many instances of a specific structure in order to improve the precision of the quantitative analysis, as demonstrated for imaging the structure of the nuclear pore complex (Szymborska *et al*., 2013).

With a view to further enhancing the speed of the SMLM workflow, particularly for automated analysis of high throughput SMLM data, the postprocessing steps may be automated, noting that the lateral drift correction step is already being applied automatically within ThunderSTORM. Further automation of the postprocessing of the localization data could be realized by setting reasonable values of the filtering and threshold parameters for a whole data set (e.g. from a multiwell plate or time series). These values could be determined manually based on a ‘quick‐view’ image that could be rapidly calculated from a subset of frames sampled from the acquired data or by utilizing phasor‐based localization. Alternatively, algorithms could be developed to rapidly search the SMLM postprocessing parameter space to optimize the final super‐resolved image, for which deep learning approaches may be appropriate (e.g. Ouyang *et al*., 2018).

As a general observation, we note that the speed of SMLM data reconstruction (and, indeed, data acquisition) can be reduced along with the data volumes by not acquiring and/or processing more image frames than is useful for the desired outcome, as there may be little additional information present in later frames where the number of localizations drops significantly. If the decrease in the number of localizations with frame number could be determined *a priori*, the SMLM data acquisition time – and the corresponding data volumes acquired – could be significantly reduced with minimal loss of information by optimizing the number of acquired frames. For high throughput SMLM, the optimization of the number of frames acquired could be set for a whole sample array or it could be determined automatically in a preview mode that rapidly samples the decrease in localizations with frame number and automatically sets the limit to the number of SMLM frames acquired. We note that techniques being developed for rapid SLM of live cells, e.g. using Bayesian analysis (Cox *et al*., 2012; Xu *et al*., 2017; Griffié *et al*., 2018), would not be suitable for ‘Parallel mode’ processing since they do not analyse sequential frames independently. However, it may be possible to adapt our ‘Batch mode approach’, where the data for each FOV could be analysed on a separate HPC node – or as a separate instance running on an HPC node.

The scripts that we run on our HPC cluster for the work presented here can be accessed at: https://github.com/ImperialCollegeLondon/HPC_STORM.

The data underlying the work presented in this manuscript can be accessed on the OMERO server at: https://omero.bioinformatics.ic.ac.uk/omero/webclient/?show=project-4802.
